# Supplementation of Heat-Treated *Lactiplantibacillus plantarum* nF1 Changes the Production of Short-Chain Fatty Acids in Healthy Infants

**DOI:** 10.1155/2024/5558566

**Published:** 2024-04-08

**Authors:** Yoowon Kwon, Kee Hyun Cho, Sangbae Ma, Hyelyun Ko, Geun-Hye Hong, So-Young Lee, Kun-Young Park, Jin A. Chung, Su Jin Jeong

**Affiliations:** ^1^Department of Pediatrics, Chungnam National University Sejong Hospital, Chungnam National University School of Medicine, Sejong, Republic of Korea; ^2^Department of Pediatrics, Kangwon National University Hospital, Kangwon National University School of Medicine, Chuncheon, Republic of Korea; ^3^AiBiotics Co Ltd, Changwon, Republic of Korea; ^4^IMMUNOBIOTECH Corp, Seoul, Republic of Korea; ^5^School of Integrated Medicine, CHA University, Seongnam, Republic of Korea; ^6^Department of Pediatrics, CHA Bundang Medical Center, CHA University School of Medicine, Seongnam, Republic of Korea

## Abstract

**Background:**

Imbalance of the gut microbiome and decrease in the number of short-chain fatty acid (SCFA)-producing bacteria often affect human health by altering intestinal and immune homeostasis. The use of probiotics has been shown to be an attractive method to modulate gut microbiota to prevent or treat intestinal dysbiosis. Likewise, this study aimed to determine whether the oral consumption of heat-treated *Lactiplantibacillus plantarum* nF1 (HLp-nF1) induces changes in the gut environment in healthy infants by measuring changes in fecal SCFAs.

**Methods:**

The study enrolled 43 infants aged under 2 months, with 30 infants in the HLp-nF1 group receiving HLp-nF1 orally (2.5 × 10^10^ cells/g/pack, daily dose of two packs) for 8 weeks. The fecal samples were collected and the questionnaires were administered at weeks 0 and 8.

**Results:**

The concentrations of the total SCFAs, acetate, propionate, and butyrate significantly increased following HLp-nF1 supplementation (*P* < 0.0001, *P* < 0.0001, *P* < 0.0001, and *P*=0.028, respectively).

**Conclusions:**

Supplementation of HLp-nF1 has a positive effect on SCFA production and could be a potentially useful and straightforward method to manipulate SCFA formation.

## 1. Introduction

The human gastrointestinal tract comprises a complex and dynamic microbial community, and reportedly, the gut microbiota is critical for numerous aspects of human health [1]. It modulates innate immunity, protects against pathogens by maintaining the intestinal mucosal barrier or providing anti-inflammatory signals to the host, and regulates metabolic homeostasis via essential nutrient synthesis and absorption [1–7].

One of the main functions of the gut microbiota is the metabolic ability to transform complex polysaccharides into simple sugars, which are fermented to form short-chain fatty acids (SCFAs) [8, 9], the main metabolites produced by the microbiota in the colon. They are defined as volatile saturated fatty acids with one to six carbon atoms in the aliphatic chain, existing in either a straight or branched conformation [9–11]. The major SCFAs produced are acetate, propionate, and butyrate [7, 12–14]. These acids need to be produced in adequate amount to maintain the gut health and the well-being of the host [9, 15]. SCFAs play important roles both directly and indirectly in regulating immune and intestinal homeostasis, provide the primary source of energy for colonocytes [16, 17], reinforce intestinal barrier function [18], regulate the immune system through various signaling pathways [9, 19–22], and control glucose or lipid metabolism [23–25].

Dysbiosis, a disturbance in the gut microbiota composition, results in a decrease in the number of bacteria producing SCFAs. In particular, infancy characterizes a highly dynamic stage wherein gut microbiota form and develop under the influence of various factors [3]. The beneficial effect of probiotics on the balance of gut microbiota and the production of metabolites, including SCFAs, has been confirmed by the results of numerous studies [9]. Recently, the interest and application of inactivated strains or dead cells, called postbiotics, have been increased due to their safety and storage capacity [26]. A postbiotic is defined as a “preparation of inanimate microorganisms and/or their components that confers a health benefit on the host” [27–30]. In the present study, we aimed to determine whether oral consumption of heat-treated *Lactiplantibacillus plantarum* nF1 (HLp-nF1) induces changes in the gut environment in healthy infants by measuring changes in fecal SCFAs.

## 2. Materials and Methods

### 2.1. Subjects

We recruited infants aged <2 months, born at the CHA Bundang Medical Center (Seongnam, Republic of Korea) and Kangwon National University Hospital (Chuncheon, Republic of Korea) between November 2021 and February 2022. The inclusion criteria were: infants born (1) between 37 and 42 weeks of gestation; (2) with a birth weight between 2,500 and 4,500 g; and (3) without any prenatal and postpartum adverse events. We excluded infants: (1) diagnosed with congenital malformations or chromosome abnormalities; (2) with a history of gastrointestinal diseases; (3) admitted after birth; (4) with a history of using systemic steroids or antibiotics; (5) exposed to foods other than breastmilk or formula; and (6) whose parents did not consent to a stool test.

This study included healthy volunteers. Infants whose parents consented to the intake of HLp-nF1 for 8 weeks were classified as the HLp-nF1 group, while the remaining infants were included in the control group.

### 2.2. Study Design

The duration of the study was 8 weeks. HLp-nF1 was manufactured by incubating *L. plantarum* nF1 for 20 h under pH control and then sterilized at 80°C for 10 min [31]. Infants in the HLp-nF1 group orally received a daily dose of two packs of HLp-nF1 (2.5 × 10^10^ cells/g/pack) (one pack in the morning and one pack in the afternoon with feeding). Infants in the control group did not receive additional experimental treatment. During the study, all subjects were instructed not to consume other probiotics or change their formula. Fecal samples and questionnaires regarding weight, height, delivery information, feeding mode, stool characteristics, and gastrointestinal symptoms were collected at weeks 0 and 8. This study was approved by the Institutional Review Board (IRB) of the Ethics Committee of CHA Bundang Medical Center (IRB no. 2021-07-077). Written informed consent was provided by the parents of infants.

### 2.3. SCFA Analysis

Fecal samples were collected from diapers using sterile swabs, immediately transferred to sterile cryogenic tubes, and stored in a −20°C freezer until delivery to the laboratory.

SCFAs were extracted from fecal samples (50 mg) using deionized water (800 *µ*L) and 5 M HCl (10 *µ*L). After brief vortexing, 400 *µ*L of ether was added to the samples, followed by mixing and shaking in the refrigerator for 5 min. After centrifugation (14,000 rpm, for 1 min), 20 *µ*L of N, O-Bis (trimethylsilyl) trifluoroacetamide was added to 200 *µ*L of ether layer, and the mixture was incubated at 70°C for 20 min and at 37°C for 2 h. Subsequently, the derivatives of SCFAs were assessed.

Gas chromatography analysis was performed using GC-2010 Plus, GCMS-TQ 8030 (Shimadzu, Tokyo, Japan) with a DB-5 ms column (inner diameter: 30 mm × 250 mm; film thickness: 0.25 *μ*m; Agilent J&W Scientific, Folsom, CA, USA). The gas chromatography conditions were as follows: 1 *µ*L of derivatives was injected in split mode with a ratio of 50 : 1; injection temperature: 200°C; and column oven temperature: 40°C. The initial temperature was 40°C and maintained for 2 min; thereafter, it was increased from 40°C to 70°C at a rate of 10°C/min, from 70°C to 85°C at a rate of 4°C/min, from 85°C to 110°C at a rate of 6°C/min, and finally from 110°C to 290°C at a rate of 90°C/min; this temperature was maintained for 6 min. Helium was used as a carrier gas at a constant flow rate of 0.89 mL/min through the column. The temperature of the electron impact ion source and interface were set to 200°C and 250°C, respectively. The detector energy was 0.1 kV, event time was 0.03 s, and the mass spectrum data were collected in scan mode (m/z 117: acetic acid, 131: propionic acid, 145: butyric acid). The concentrations of SCFAs were calculated using a standard solution of butyric acid (B103500; Sigma-Aldrich Co., St. Louis, MO, USA), propionic acid (94425; Sigma-Aldrich Co.), and acetic acid (31010S0350; JUNSEI Chemical Co., Tokyo, Japan).

### 2.4. Statistical Analysis

Data were analyzed using descriptive statistics and presented as means with standard deviations, or as medians with an interquartile range (IQR), and proportions. Comparisons of the mean values of continuous and categorical variables between groups were conducted using the *t*-test and Fisher's exact-test, respectively. Linear regression coefficient analyses were used to calculate unstandardized regression coefficients. *P* values <0.05 de-noted statistically significant differences. All statistical analyses were performed using IBM SPSS® Statistics 28.0.1.1 (IBM Corporation, Armonk, NY, USA).

## 3. Results

### 3.1. Comparison of Baseline Characteristics between Control and HLp-nF1 Groups

Forty-three healthy volunteers were included in this study. The number of subjects included in the control and HLp-nF1 groups was 13 and 30, respectively. Baseline characteristics, including sex, birth information, feeding mode, and age, did not vary significantly between the two groups (Table 1).

### 3.2. Comparison of Questionnaire Data after 8 Weeks between Control and HLp-nF1 Groups

In this study, there were no cases of withdrawal due to side effects or loss at follow-up. Hence, a total of 43 subjects completed the questionnaire after 8 weeks. There was no significant difference between the two groups in any of the questionnaire items (Table 2). Mean weight and height after 8 weeks were 6,200 g and 62.04 cm in the control group, and 6,285.18 g and 60.57 cm in the HLp-nF1 group, respectively. Weight and height growth assessment was properly performed in both groups. More than half of the cases did not report gastrointestinal symptoms. Defecation was more frequent in the HLp-nF1 group versus the control group, but the difference was not statistically significant.

### 3.3. Concentration of SCFAs and Effect of HLp-nF1 Supplementation

**Figure 1:**
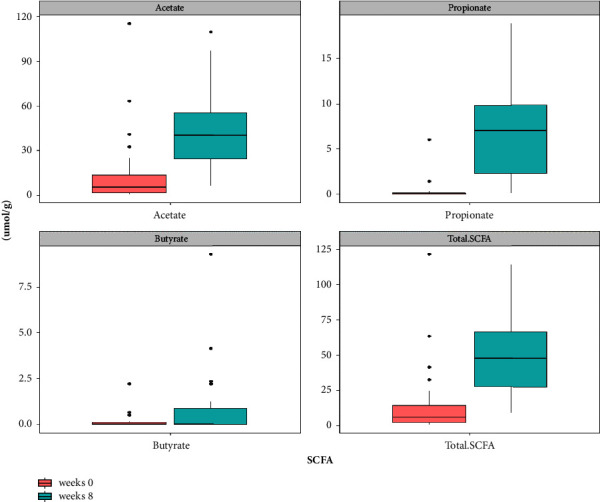
Box plots of the concentrations of SCFAs in the HLp-nF1 group.

We performed two measurements of SCFA metabolites at weeks 0 and 8 to observe changes that occurred during the supplementation of HLp-nF1. Acetate was the most abundant SCFA, followed by propionate and butyrate. The concentrations of total SCFAs, acetate, propionate, and butyrate were significantly increased after HLp-nF1 supplementation (*P* < 0.0001, *P* < 0.0001, *P* < 0.0001, and *P*=0.028, respectively). Although the same trend was observed in the control group, the results were not statistically significant (Table 3, Figure 1).

Linear regression coefficient analyses of differences in the concentration of SCFAs in association with other variables in the HLp-nF1 group were performed to investigate the effect of HLp-nF1 supplementation. Other variables (i.e., sex, age, birth mode, birth weeks, birth weight, birth height, birth head circumference, and feeding mode) were not significantly associated with changes in SCFA concentration, except for birth weeks at the propionate analysis (Table 4).

## 4. Discussion

In this study, we compared changes in the gut environment of healthy infants after the administration of HLp-nF1 for 8 weeks by monitoring clinical response and fecal SCFAs. First, adequate growth was observed in both groups, and safety was demonstrated. Second, SCFA production was significantly increased in the HLp-nF1 group compared with the control group.

In terms of clinical response, there were no cases of withdrawal due to side effects or gastrointestinal symptoms reported during the study period. Growth, the most important factor in infants, was appropriate in terms of height and weight. Safety is one of the most important criteria in the selection of probiotic strains for human consumption. In particular, HLp-nF1 is nonviable heat-killed microorganism, which exhibits a low risk of sepsis or bacteremia associated with probiotics [32–37], particularly in critically ill or vulnerable patients and pediatric populations, demonstrating comparable effects to those of viable probiotics [26, 36, 38–40].

Acetate, propionate, and butyrate account for 85%–95% of the total SCFAs in all regions of the colon [9, 41]. Notably, acetate is the most abundant SCFA, accounting for >50% of the total SCFAs [9, 42]. In accordance with previous studies, the concentration of acetate was the highest among all SCFAs in this study. However, the baseline values of fecal SCFAs and the different age- or diet-related patterns of change in SCFAs remain unknown [43]. In one study measuring fecal SCFAs at birth, day 30, and day 60 in healthy term infants from different feeding groups (i.e., extensively hydrolyzed formula, amino acid formula, or human milk), there was no significant trend with increasing age up to day 60. In the human milk group, the average concentration of total SCFAs at birth, day 30, and day 60 was approximately 40 mmol/g, similar to that of acetate; the average concentration of propionate and butyrate was <2 *μ*mol/g [44]. These values were similar to the SCFA concentration measured in the control group at week 8 in our study. While both groups showed an increasing trend in SCFA concentrations after 8 weeks, the difference was not statistically significant in the control group. Furthermore, other factors that could affect the SCFA concentration were evaluated by performing linear regression coefficient analyses. The results of these analyses confirmed the relationship between differences in the concentration of SCFAs and other variables, including sex, age, birth mode, birth weeks, birth weight, birth height, birth head circumference, and feeding mode.

It has been shown that SCFAs play a crucial role in maintaining intestinal and immune homeostasis, particularly in regulating the maturation, integrity, and function of the gut barrier [9, 43]. After supplying colonocytes, SCFAs are transported from the intestinal cavity into the blood vessels and finally to organs as substrates or signaling molecules to perform numerous physiological functions [25]. In addition, since 95% of SCFAs are reabsorbed or metabolized by gut microbiota, small changes in SCFA concentration in fecal excretion actually represent large differences in production in the gut [12]. Therefore, the significant increase in SCFAs recorded after 8 weeks of HLp-nF1 intake can be considered meaningful.

While SCFA is influenced by multiple factors, the most significant mechanism inducing a meaningful change in SCFA concentrations following HLp-nF1 consumption can be attributed to alterations in the composition of gut microbiota. In the microbiome analysis of 30 infants within the HLp-nF1 group, altered bacterial composition after HLp-nF1 intake was observed; marked increases were noted in beneficial bacteria, such as *Bifidobacterium* and *Veillonella*, while decreases were observed in certain opportunistic pathogens, including *Prevotella*, *Streptococcus*, and *Sutterella* (Figure S1, Table S1). Although a comprehensive analysis of gut microbiota composition was not conducted in this study, we hypothesize that ingestion of HLp-nF1 may impact rebiosis, the re-establishment of the native microbiota, by serving as a barrier and impeding the colonization of opportunistic bacteria [45]. Especially, *Lactiplantibacillus* strains enhance the integrity of the intestinal barrier, which may affect decreasing translocation of bacteria across the intestinal mucosa [46]. One possible reason for the absence of an increase in the relative abundance of *Lactiplantibacillus* per se is thought to be the low quantity and short duration of HLp-nF1 administered relative to the total gut microbes. Nevertheless, this regulation of the gut environment induced by the supplementation of HLp-nF1 may have affected the increase in SCFAs by controlling the ratio of beneficial bacteria to opportunistic pathogens. Previous studies on *L. casei* or *L. plantarum* also showed similar alterations in the composition of gut microbiota, with changes in SCFAs [12, 47, 48]. Furthermore, SCFAs are produced by different bacterial species possessing specific enzymes [49]. The two genera whose relative abundance increased in this study, *Bifidobacteria* and *Veillonella,* are established SCFA-producing bacteria. *Bifidobacteria* mainly produce acetate and formate through the fermentation pathway under carbohydrate limitation. They also produce acetate and lactate when carbohydrates are in excess through the pentose phosphate pathway [14, 15, 49, 50]. *Veillonella* produces propionate through the succinate pathway [51–53]. Human milk oligosaccharides in breastmilk and galacto-oligosaccharides or fructo-oligosaccharides in infant formula possess prebiotic properties that allow these bacteria to effectively produce SCFAs [54, 55]. In addition to these mechanisms, exopolysaccharides (EPS) (the polysaccharides synthesized and secreted by bacteria) may also be a major source of SCFAs. *Lactiplantibacillus* and *Bifidobacterium* are the main EPS-producing strains, and they can degrade and ferment EPS into SCFAs [56, 57].

A couple of study limitations should be noted. Firstly, the number of subjects in the control group was relatively small. Because the study was conducted with healthy infants who met various conditions, there was difficulty in recruiting a sufficient number of subjects. Secondly, it was difficult to control all factors affecting the concentration of SCFAs. The concentration and ratio of SCFAs are associated with the composition of the gut microbiome, diet, genetics, and other environment factors [9, 48]. However, subjects of a similar age who had a restrictive diet before initiating feeding with solid food were recruited. Factors that could exert an effect, such as birth mode, were similar between the two groups.

Nevertheless, the results of this study are significant because we confirmed that postbiotics intake for a short period of 8 weeks in infancy, when alterations in the gastrointestinal environment are very dynamic, induces changes in the production of SCFAs. This study is also meaningful in that it is the first study of changes in SCFAs using HLp-nF1 to the best of our knowledge. Further research and long-term follow-up studies in various species and numerous strains are warranted to better understand the effects of postbiotics administration on the production of specific SCFAs.

## 5. Conclusions

This study demonstrated that an 8-week oral administration of HLp-nF1 may increase SCFA production in healthy infants. It can be suggested that the use of postbiotics is a useful and easy method for manipulating SCFA formation by alterations in the human gut microbiota. This approach can be utilized to prevent or treat intestinal dysbiosis.

## Figures and Tables

**Table 1:** Baseline characteristics of children in the control and HLp-nF1 groups.

	*n* or Mean ± SD		*P* value
	Control group (*n* = 13)	HLp-nF1 group (*n* = 30)	
Sex			
Female	7 (53.8)	13 (43.3)	1.00
Male	6 (46.2)	17 (56.7)	
Birth mode			
NSVD	3 (23.1)	8 (26.7)	0.667
C/S	10 (76.9)	22 (73.3)	
Birth weeks	38 (1/7) ± 1.05	38 (4/7) ± 0.82	0.15
Birth weight (g)	3,045.77 ± 429.10	3,111.33 ± 285.32	0.56
Birth height (cm)	48.91 ± 2.51	48.95 ± 1.53	0.96
Birth head circumference (cm)	33.83 ± 1.16	34.23 ± 1.20	0.31
Feeding mode			
Formula	10 (76.9)	21 (70.0)	0.689
Mixed	3 (23.1)	9 (30.0)	
Age (days)	14.92 ± 14.16	21.13 ± 22.27	0.361

Data are presented as numbers (percent) or means ± SD. Comparisons of mean values of continuous and categorical variables between groups were conducted using the *t*-test and Fisher's exact-test, respectively; *P* values <0.05 denote statistically significant differences. C/S, cesarean section; HLp-nF1, heat-treated *Lactiplantibacillus plantarum* nF1; NSVD, normal spontaneous vaginal delivery; SD, standard deviation.

**Table 2:** Questionnaire after 8 weeks in the control and HLp-nF1 groups.

	*n* or Mean ± SD		*P* value
	Control group (*n* = 13)	HLp-nF1 group (*n* = 30)	
Weight (g)	6,200.0 ± 1,105.29	6,285.18 ± 872.45	0.858
Height (cm)	62.04 ± 6.76	60.57 ± 3.50	0.321
Gastrointestinal symptoms			
Vomiting	1 (7.7)	2 (6.7)	0.361
Loose stool or diarrhea	0 (0.0)	3 (10.0)	
Constipation	2 (15.4)	1 (3.3)	
Poor weight gain	0 (0.0)	2 (6.7)	
Irritability	1 (7.7)	2 (6.7)	
None	9 (69.2)	20 (66.7)	
Stool frequency (per day)			
<1	5 (38.5)	5 (16.7)	0.254
1	5 (38.5)	8 (26.7)	
2-3	3 (23.0)	12 (40.0)	
>3	0 (0.0)	5 (16.7)	

Data are presented as numbers (percent) or means ± SD. Comparisons of the mean values of continuous and categorical variables between groups were conducted using the *t*-test and Fisher's exact-test, respectively; *P* values <0.05 denote statistically significant differences. HLp-nF1, heat-treated *Lactiplantibacillus plantarum* nF1; SD, standard deviation.

**Table 3:** Changes in SCFA concentration in the control and HLp-nF1 groups.

	SCFA concentration (*μ*mol/g)		
	Week 0	Week 8	*P* value
	Control group (*n* = 13)		

Total^*∗*^	13.511 ± 22.95	26.067 ± 28.04	0.270
Acetate	12.802 ± 22.35	21.557 ± 25.81	0.471
Propionate	0.541 ± 1.03	3.976 ± 3.63	0.111
Butyrate	0.168 ± 0.44	0.534 ± 0.71	0.138

	HLp-nF1 group (*n* = 30)		

Total	14.398 ± 24.146	50.157 ± 26.761	<0.0001
Acetate	13.898 ± 23.232	42.185 ± 24.401	<0.0001
Propionate	0.347 ± 1.084	7.157 ± 5.109	<0.0001
Butyrate	0.153 ± 0.407	0.816 ± 1.822	0.028

^*∗*^Total SCFAs refers to the sum of acetate, propionate, and butyrate. Data are presented as means ± SD. *P* values <0.05 denote statistically significant differences. HLp-nF1, heat-treated *Lactiplantibacillus plantarum* nF1; SCFA, short-chain fatty acid.

**Table 4:** Linear regression coefficients analyses of differences in the concentration of SCFAs in association with other variables in the HLp-nF1 group.

Variable	Acetate		Propionate		Butyrate	
	B	*P* value	B	*P* value	B	*P* value
Sex	22.440	0.173	4.174	0.096	−0.187	0.843
Age	−0.046	0.912	−0.038	0.544	0.019	0.448
Birth mode	14.612	0.372	1.245	0.611	1.094	0.256
Birth weeks	−2.300	0.813	−3.960	0.012	0.294	0.608
Birth weight (g)	0.000	0.994	0.004	0.460	−0.004	0.109
Birth height (cm)	0.307	0.958	−0.555	0.527	0.411	0.234
Birth head circumference (cm)	6.631	0.330	−0.625	0.539	0.439	0.272
Feeding mode	−0.256	0.988	2.347	0.347	−1.430	0.147

B denotes unstandardized regression coefficients. *P* values <0.05 denote statistically significant differences. HLp-nF1, heat-treated *Lactiplantibacillus plantarum* nF1; SCFA, short-chain fatty acid.

## Data Availability

The raw data presented is this study is openly available in the Zenodo repository under the following link https://doi.org/10.5281/zenodo.8267872. These datasets include raw experimental data and further inquiries are available from the corresponding author upon reasonable request.

## Supplementary Materials

Supplementary Materials Methods and results about microbiome analysis are included in the supplementary material (Figure S1, Table S1, and Method).
