# Supplementary material for: Supplementation of Heat-Treated Lactiplantibacillus plantarum nF1 Changes the Production of Short-Chain Fatty Acids in Healthy Infants
Source: J Nutr Metab. 2024 Apr 8;2024:5558566. doi: 10.1155/2024/5558566 (PMC11018375; doi:10.1155/2024/5558566)
Supplement: Supplementary Materials — Methods and results about microbiome analysis are included in the supplementary material (Figure S1, Table S1, and Method). [file 5558566.f1.pdf]

Supplementary Material

Additional files

Figure S1. Gut microbiota composition in the HLp-nF1 group at the genus level.

Table S1. Abundance ratios of gut microbiota composition in the HLp-nF1 group at the genus level.

Methods Supplement – Microbiome Analysis

Figure S1. Gut microbiota composition in the HLp-nF1 group at the genus level.

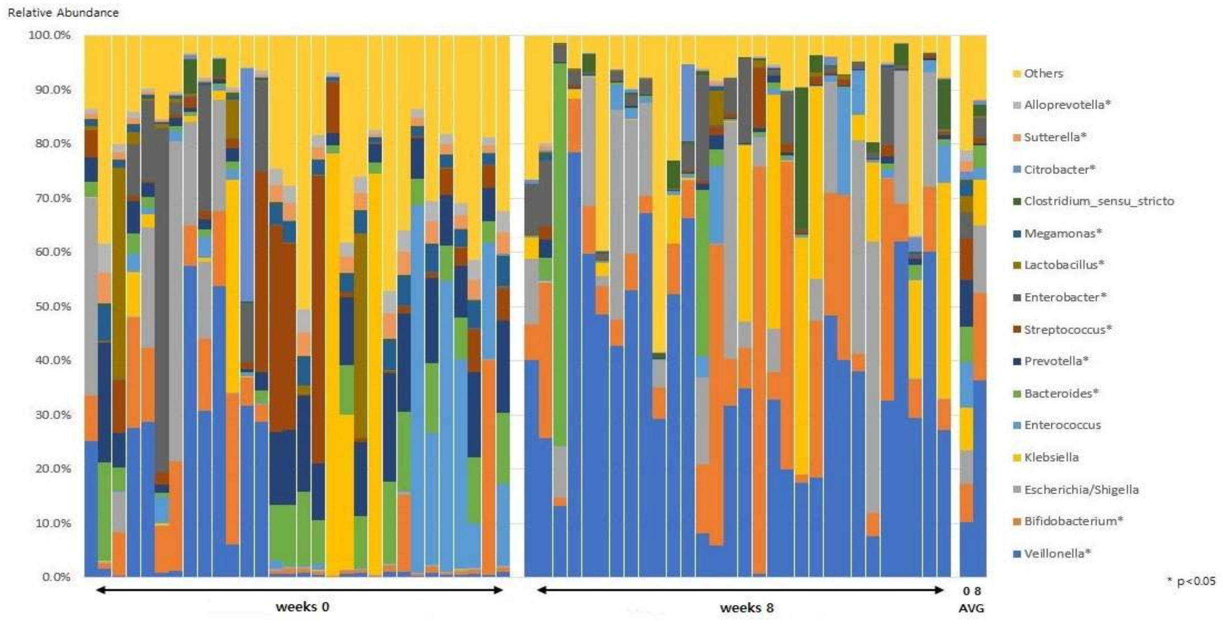

Table S1. Abundance ratios of gut microbiota composition in the HLp-nF1 group at the genus level.

Genus	Abundance ratio (%)		
	**Week 0**	**Week 8**	**P-value**
Veillonella	0.841 (0.544–25.810)	33.824 (19.488–52.385)	<0.001
Bifidobacterium	1.128 (0.607–13.291)	7.328 (5.309–24.009)	0.004
Escherichia/Shigella	0.324 (0.138–2.193)	6.557 (0.093–21.767)	0.152
Klebsiella	0.006 (0.044–1.254)	0.183 (0.024–10.415)	1.0
Enterococcus	0.951 (0.116–5.740)	0.517 (0.188–1.907)	0.734
Bacteroides	4.656 (1.487–11.404)	0.255 (0.146–0.954)	<0.001
Prevotella	6.934 (2.330–14.276)	0.165 (0.108–0.782)	<0.001
Streptococcus	1.661 (0.438–6.297)	0.368 (0.103–0.856)	<0.001
Enterobacter	0.128 (0.044–0.937)	1.081 (0.188–5.221)	0.023
Lactiplantibacillus	0.093 (0.053–0.747)	0.048 (0.021–0.245)	0.036
Megamonas	1.737 (0.597–4.280)	0.039 (0.027–0.214)	<0.001
Clostridium	0.200 (0.122– 0.297)	0.151 (0.097–1.072)	0.79
Citrobacter	0.032 (0.017– 0.199)	0.117 (0.045–0.168)	0.033
Sutterella	1.494 (0.443–3.345)	0.031 (0.023–0.129)	<0.001
Alloprevotella	1.509 (0.502–3.312)	0.031 (0.019–0.158)	<0.001

Data are presented as median (IQR). P-values <0.05 denote statistically significant differences. HLp-nF1, heat-treated *Lactiplantibacillus plantarum* nF1.

At the genus level, the relative abundance of *Veillonella* and *Bifidobacterium* exhibited a statistically significant increase ($P < 0.001$ and $P = 0.04$, respectively) following HLp-nF1 supplementation. In contrast, the abundance of *Bacteroides, Prevotella, Streptococcus, Lactiplantibacillus, Megamonas, Sutterella,* and *Alloprevotella* was significantly decreased ($P < 0.001$, $P < 0.001$, $P < 0.001$, $P = 0.036$, $P < 0.001$, $P < 0.001$, and $P < 0.001$, respectively).

Methods Supplement – Microbiome Analysis

Fecal samples were collected from diapers using feces sampling kit spoon type (No-ble Biosciences, Hwaseong, Republic of Korea) and stored in a storage buffer. Samples were stored at −80°C until DNA extraction within 48 h from the time of collection.

For the extraction of total genomic DNA from fecal samples, 500 μL of pre-treatment solution (TianLong Science and Technology Co., Xi'an, China) was added to the samples. Samples were vortexed for 1 min, incubated at 80℃ for 10 min, and centrifuged at 14,000 rpm for 1 min. DNA was extracted using a nucleic acid extraction kit (Stool DNA/RNA Extraction Kit; TianLong Science and Technology Co.) by injecting 10 μL of proteinase K into 200 μL of the supernatant. Next, the concentration and purity of the extracted DNA were measured with a DNA/protein Analyzer (Pultton Tehcnology Ltd., San Jose, CA, USA).

PCRBIO VeriFi Mix (PCR Biosystems Ltd., London, UK) and primers (Macrogen, Seoul, Republic of Korea) were used to amplify the 16S rRNA V3-V4 region of the extracted DNA. The V3-V4 target region forward primer (5′TCGTCGGCAGCGTCAGA TGTG-TATAAGAGACAGCCTACGGGNGGCWGCAG3′) and reverse primer (5′GTCTCGTGGGCTCGGAGATGTGTATAAGAGACAGGACTACHVGGGTATCTAATCC′3) were used according to the guideline 'Preparing 16S Ribosomal RNA Gene Ampli-cons for the Illumina MiSeq System' (Part#15044223 Rev.B; Illumina, San Diego, CA, USA). The reaction conditions in the PCR Thermal Cycler (TianLong Science and Technology Co.) were as follows: 95°C for 3 min; 25 cycles at 95°C for 30 s; 55°C for 30 s; and 72°C for 30 s, with a final extension at 72°C for 5 min. The PCR products were purified in Nucleic Acid Extracter (TianLong Science and Technology Co.) using a MagListo™ PCR/Gel Purifica-tion Kit (Bioneer, Daejeon, Republic of Korea).

After PCR cleanup, secondary amplification was performed through the attachment of a dual index using Nextra XT Index kit v2 Set A and Set B (Illumina) in a PCR Thermal Cycler (TianLong Science and Technology Co.) as follows: 95°C for 3 min; eight cycles at 95°C for 30 s; 55°C for 30 s; and 72°C for 30 s, with a final extension at 72°C for 5 min. The PCR products were purified in Nucleic Acid Extracter (TianLong Science and Technology Co.) using a MagListo™ PCR/Gel Purification Kit (Bioneer). The size of the purified DNA products was measured using Qsep 100 (Bioptic, Changzhou City, China), and the quality was evaluated on a Qubit Flex fluorometer (Thermo Fisher, Waltham, MA, USA).

The concentration of each DNA library was adjusted to 4 nM, and all libraries were pooled into a single mixture. The size and concentration of the final pooled libraries were determined. The pooled libraries were denatured with 0.2 N NaOH, diluted with hybridi-zation buffer from the Illumina MiSeq Reagent Kit v3 600-cycle, injected into the kit v3 car-tridge, and loaded into the Illumina MiSeq instrument for sequencing (Illumina). The PhiX Control v3 kit (Illumina) was used as internal control. Analysis of the 16S rRNA gene se-quences was performed using the 16S metagenomics app (database: RefSeq Ribosomal Database Project [RDP] 16s DADA2), platform BaseSpace Sequence Hub (Illumina).
